# Effect of Ammonium Salt on Conjugated Polyelectrolyte as an Interlayer for Organic–Inorganic Hybrid Perovskite Memristors

**DOI:** 10.3390/nano15030227

**Published:** 2025-01-30

**Authors:** Eun Soo Shim, Ji Hyeon Lee, Ju Wan Park, Sun Woo Kim, Su Bin Park, Jea Woong Jo

**Affiliations:** Department of Energy and Materials Engineering, Dongguk University, 30 Pildong-ro, 1-gil, Jung-gu, Seoul 04620, Republic of Korea

**Keywords:** conjugated polyelectrolyte, ammonium salt, organic–inorganic hybrid perovskite, memristor, interlayer

## Abstract

Memristors are promising candidates for next-generation non-volatile memory devices, offering low power consumption and high-speed switching capabilities. However, conventional metal oxide-based memristors are constrained by fabrication complexity and high costs, limiting their commercial viability. Organic–inorganic hybrid perovskites (OIHPs), known for their facile solution processability and unique ionic–electronic conductivity, provide an attractive alternative. This study presents a conjugated polyelectrolyte (CPE), PhNa-1T, as an interlayer for OIHP memristors to enhance the high-resistance state (HRS) performance. A post-treatment process using *n*-octylammonium bromide (OABr) was further applied to optimize the interlayer properties. Devices treated with PhNa-1T/OABr achieved a significantly improved ON/OFF ratio of 2150, compared to 197 for untreated devices. Systematic characterization revealed that OABr treatment improved film morphology, reduced crystallite strain, and optimized energy level alignment, thereby reinforcing the Schottky barrier and minimizing current leakage. These findings highlight the potential of tailored interlayer engineering to improve OIHP-based memristor performance, offering promising prospects for applications in non-volatile memory technologies.

## 1. Introduction

Memristors are regarded as promising random access memory devices with a simple sandwiched metal–insulator–metal (MIM) architecture, low power consumption, and fast switching capability [1,2,3]. However, conventional memristic metal oxide materials such as titanium, tantalum, and hafnium oxides suffer from uneconomical and complicated fabrication processing steps, limiting the commercialization of the memristors despite the fascinating memristic performances. For instance, metal oxides typically require high-temperature processing (>300 °C) for complete oxidation along with high vacuum-assisted depositions, increasing the manufacturing costs. Furthermore, the intrinsic robustness of metal oxides limits their application in deformable memristors for next-generation electronics. In analogue, organic materials are also a great potential as form factor-free memristors and biomimetics [4,5,6,7]. Accordingly, organic–inorganic hybrid perovskites (OIHPs), which exhibit facile solution processability, have been recognized as a superior candidate for the next-generation memristic material [8,9,10]. Although most of the focus for utilization of OIHPs has been implicated in photovoltaics [11,12], photodetectors [13,14], and transistors [15], OIHPs are capable of being a superior active memristic material due to their low ion migration energy (<1.0 eV), simple compositional engineering (1.2–3.6 eV), and desirable hysteresis derived from the unique mixed ionic–electronic conductivity [16,17,18,19,20]. Accordingly, OIHP-based memristors are great candidates for neuromorphic computing, artificial synapse, and vision processing applications [21,22].

Memristors fundamentally require two different resistances at a common voltage. A large gap between the high resistance state (HRS) and low resistance state (LRS) is a prerequisite for an accurate reading of saved data, which is crucial when incorporated in an array set-up [23]. Electrical biasing of OIHPs with electroactive metals such as silver (Ag) and aluminum (Al) induces charge filament (CF) formations within OIHP layers through metal ion migration, allowing direct ohmic conduction between the electrodes in MIM configuration [24]. CFs allow securing the low value of resistance at LRS to be more straightforward than the high resistance at HRS. Regulating defect density within the OIHPs [25], passivating the OIHP surface [26], and interlayers [27] have been explored to enhance the resistance at HRS for OIHP memristors. Among these approaches, the selection of suitable interlayers is known to synergistically produce charge-selective Ohmic contact with an electrode and engineer the bulk defect density of OIHPs [28,29].

Key functionalities for the selective charge collection as an interlayer rely on the type of majority carrier density, energy level alignment, and dipole regulation [30,31]. In effect, organic-based interlayers are advantageous to meet the economic feasibility and mechanical flexibility of next-generation electronics [32]. Recently, conjugated polyelectrolytes (CPEs), which are special derivatives of conjugated polymers with ionic groups in side chains, have emerged as promising interlayer materials for perovskite electronics. Particularly, its ionic pendant groups provide CPEs to possess increased conductivity via self-doping, energy level modulation through dipole inducement, and higher surface energy [33,34,35,36,37]. Increased hydrophilicity from the ionic functionality for CPE is additionally beneficial for the growth of OIHPs in solution processing due to the polar nature of OIHPs [38].

Here, we adopted the CPE, PhNa-1T, as an interlayer between an electrode and the OIHP for the first time in memristor applications. Moreover, the additional post-treatment with alkylammonium salt on the CPE interlayer, which would improve the resistance at the HRS by reducing the doping of CPE, was applied. Moreover, the OIHP layer on OABr-treated CPE was found to have more favorable crystallographic characteristics due to the alkylammonium and lead interaction. As a result, the OIHP memristor with CPE interlayer after alkyl ammonium salt treatment was demonstrated to provide an increased ON/OFF ratio of 2150 from 197. Step-by-step thin film characterization revealed that the post-treatment of CPE is effective at formulating a more favorable OIHP layer. Moreover, a desirable Schottky junction was formed between the electrodes, which was found to be effective at suppressing current flow before the conductive filament formation within OIHP memristors.

## 2. Materials and Methods

### 2.1. Materials

Methyl ammonium bromide (MABr), phenethyl ammonium bromide (PEABr), and *n*-octyl ammonium bromide (OABr) were purchased from Greatcell Solar Materials. Lead (II) bromide (PbBr_2_) was purchased from Tokyo Chemical Industry. *N*,*N*-Dimethylformamide (DMF), diemthy sulfoxide (DMSO), and 2-propanol were purchased from Sigma-Aldrich. All the unspecified materials are provided by Sigma-Aldrich.

### 2.2. Preparation of Organic–Inorganic Hybrid Perovskite Memristors

Indium-doped tin oxide (ITO) (15 mm × 15 mm) substrates were cleaned with soapy water, deionized water, acetone, and 2-propanol successively through water bath sonication for 15 min each. Cleaned ITOs were blown with N_2_ gun and left in convection oven at temperature of 100 °C for 20 min. Dried substrates were then treated under UV–Ozone treatment for 20 min before spin-coating processes. For PhNa-1T deposition, 2 mg mL^−1^ of PhNa-1T in deionized water was prepared and filtered. Filtered solutions were mixed with 2-propanol at 1:1 *v*/*v* and spin-coated on the ITO substrates with spinning rate of 3000 rpm for 60 s. The films were left on a hotplate at 100 °C for 10 min. An amount of 20 mg mL^−1^ of OABr in 2-propanol was spin-coated on the PhNa-1T to prepare PhNa-1T/OABr interlayer. To prepare OIHP layer, 1.4 M of PbBr_2_ in DMF:DMSO (8:1 *v*/*v*) was spin-coated on the substrate at 4000 rpm for 30 s, which would provide a thickness of ~550 nm [39]. Then, 20 mg mL^−1^ of MABr:PEABr (1:1 *w*/*w* in 2-propanol) was dynamically spin-coated at 4000 rpm for 30 s and left on a hotplate for 10 min at 100 °C. For passivation of the OIHPs, 2 mg mL^−1^ of OABr in 2-propanol was spin-coated on the OIHP layer at 4000 rpm for 30 s with further annealing on a hotplate at 100 °C for 10 min. The growth of low dimensional OIHP was reported to be optimized at 100 °C [40]. All the processes were performed under ambient conditions (relative humidity of 25–35% and temperature of 25–35 °C). Lastly, the samples were transferred into high vacuum chamber (<10^−7^ torr) with patterned aluminum shadow mask (d = 100 μm) to thermally evaporate Ag (100 nm).

### 2.3. Characterization

Ultraviolet–visible (UV-Vis) absorbance spectra were obtained by using V-770 (JASCO, Tokyo, Japan). Atomic force microscopy (AFM) and Kelvin probe force microscopy (KPFM) images were taken with Multimode8 (Bruker, Pittsfield, MA, USA). X-ray photoelectron spectroscopy (XPS) and ultraviolet photoelectron spectroscopy (UPS) were performed with Versaprobe II (ULVAC-PHI, Kanagawa, Japan). X-ray diffraction (XRD) was taken with Ultima IV (Rigaku, Tokyo, Japan). Electrical characterizations were performed with Keithly 4200-SCS (Keithly, Cleveland, OH, USA) connected to probe station (MS tech, Seoul, Republic of Korea).

## 3. Results and Discussions

**Figure 1:**
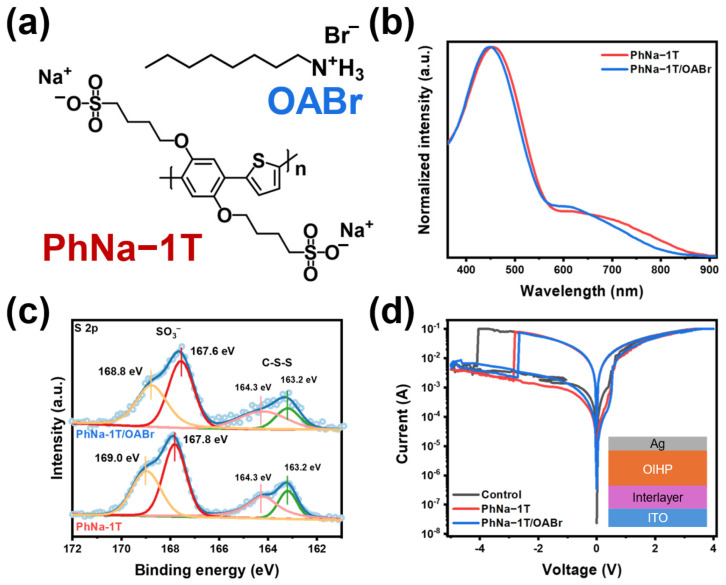
(**a**) Chemical structure of PhNa−1T and OABr. (**b**) UV−Vis absorbance spectra of PhNa−1T and PhNa−1T/OABr. (**c**) XPS plots of S 2p for PhNa−1T films before and after OABr treatment (dotted points are raw data, and blue lines are total fitted data, yellow lines are S 2*p*_1/2_ for SO_3_^−^, red lines are S 2*p*_3/2_ for SO_3_^−^, peach lines are S 2*p*_1/2_ for S, and green lines are S 2*p*_3/2_ for S). (**d**) Memristic *I−V* curve of PhNa−1T interlayer with OIHP (inset image: device architecture).

The chemical structure of CPE, named PhNa-1T, and OABr ammonium salt are shown in Figure 1a. PhNa-1T has the anionic sulfonated pendant group (SO_3_^−^) on the phenyl-thiophene copolymeric conjugated backbone. The anionic sulfonate group is known to generate polarons along conjugated polymers through electrostatic interaction [41]. OABr ammonium salt was chosen due to the high passivating capability for OIHPs as previously reported [42,43]. Moreover, ammonium salt was reported to have a strong interaction with SO_3_^−^ in the CPE [44]. Figure 1b shows absorbance spectra in the film state of PhNa-1T and PhNa-1T/OABr, in which the polaron states are observed (λ = 600–800 nm). Note that PhNa-1T/OABr samples were prepared by spin-coating OABr solution, dissolved in 2-propanol, onto PhNa-1T films whereas PhNa-1T is insoluble in 2-propanol. The maximum absorbance peak at a wavelength of 454 nm and the peak was shown to be blue-shifted to 447 nm when treated with OABr. We attribute this to the variation in the planarity of the CPE backbone after the OABr treatment, which possibly causes a partial ion exchange from sodium ion (Na^+^) to *n*-octyl ammonium [45].

In addition, the magnitude of the self-doping effect was also affected by the ion exchange, as evidenced by the diminished UV-vis absorption intensity in the wavelength region of 700–800 nm [46]. The effect of ion exchange is observed from XPS measurement of S 2*p* as shown in Figure 1c. The S 2*p* XPS data showed two distinct peaks assigned for SO_3_^−^ at higher binding energy and C-S-C at lower energy regions [47]. The peaks were deconvoluted and it was found that the peaks assigned for C-S-C showed insignificant differences after the OABr treatment, showing constant peaks at 163.2 and 164.3 eV for S 2*p*_1/2_ and S 2*p*_3/2_, respectively. However, the peaks corresponding to SO_3_^−^ for PhNa-1T at 167.8 and 169.0 eV showed the peak shifts to a lower binding energy at 167.6 and 168.8 eV (−0.2 eV). The shift to the lower binding energy of sulfur from sulfonate results from a weaker electronegativity of ammonium compared to that of sodium ion.

The PhNa-1T and PhNa-1T/OABr were then applied at the interface between OIHP and the bottom electrode (BE). ITO was used for the BE, and the top electrode (TE) was prepared with Ag. OIHP was prepared through a two-step deposition [48]. The current–voltage (*I*–*V*) data are shown in Figure 1d. The voltage scan steps at the TE were as follows: 0 V→ +4 V→ −5 V → 0 V. The memristic behavior was observed with the device configuration (inset image of Figure 1d) with similar LRS current values of 2.92 × 10^−3^, 2.82 × 10^−3^, and 2.91 × 10^−3^ A for control, PhNa-1T, and PhNa-1T/OABr at 0.1 V, respectively. The drop of current values at HRS (0.1 V) was observed from 2.19 × 10^−4^ A (control) to 5.72 × 10^−5^ A with PhNa-1T, and 3.23 × 10^−5^ A with PhNa-1T/OABr. The ON/OFF ratios for bare ITO, PhNa-1T, and PhNa-1T/OABr devices were calculated to be 13.3, 49.3, and 90.1. The initial assessment for enhanced resistance level at HRS was compared with the conductance of PhNa-1T and the PhNa-1T/OABr interlayer. The *I*–*V* data acquired from the device architecture of ITO/Interlayer/Ag is shown in Figure S1. The resistance value of ITO/Ag was 1 Ω while the value of ITO/PhNa-1T/Ag was 12.5 Ω, and ITO/PhNa-1T/OABr/1T was 21.9 Ω (active area of 0.091 cm^−2^). We ascribe the reduced conductance after inserting CPE between BE and TE to the enhanced resistance value at HRS of OIHP memristors. Moreover, deducting the polaron state of the CPE with the OABr treatment would reduce the charge transport ability of CPE and thus result in obtaining a higher resistance at HRS [49,50].

**Figure 2:**
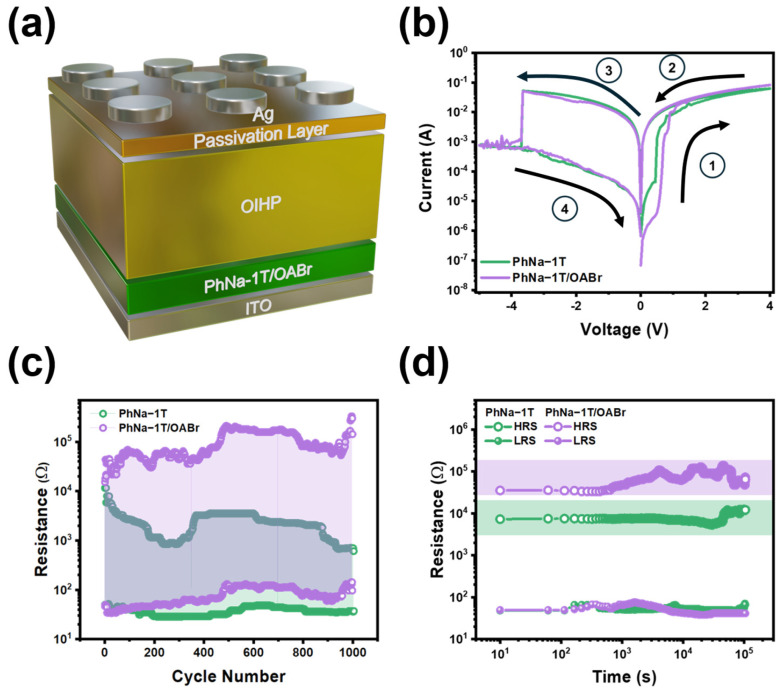
(**a**) Schematic device configuration of the OHP memristor used in this study. (**b**) Current–voltage measurement of the memristors with PhNa−1T and PhNa−1T/OABr interlayers. (**c**) Endurance and (**d**) retention testing results for OIHP memristors depending on interlayers (Resistance range at HRS were shaded with color for retention tests).

To further verify the effectiveness of OABr treatment on the CPE interlayer in OIHP memristors, a surface passivation strategy was adopted for the OIHP layer [51]. Surface passivation of the OIHP layer was performed to improve the LRS/HRS resistance ratio and operational stability. The schematic device architecture of the memristors is illustrated in Figure 2a. The representative *I–V* curves of the memristors with PhNa-1T and PhNa-1T/OABr interlayers are shown in Figure 2b. The first *I–V* scans of the memristors showing a steep increment of current upon biasing, which is an indication of electro-forming of Ag-based conductive filaments, are shown in Figure S2. The current compliance of 0.1 A was used. The CF conduction mechanism, as revealed by ohmic (n ~ 1), trap-related (n > 2), and trap-free (n ~ 2) space charge, limited current regions during SET, and the RESET process is observed, as shown in Figure S3. The current values at the HRS for the PhNa-1 T-based memristors were measured at an electrical bias of 0.1 V was 8.18 × 10^−6^ A, whereas the PhNa-1T/OABr device resulted in a current value of 7.59 × 10^−7^ A. The current values at LRS were both 1.61 × 10^−3^ and 1.63 × 10^−3^ A for the PhNa-1T and PhNa-1T/OABr. The corresponding ON/OFF ratio for the PhNa-1T- and PhNa-1T/OABr-based OIHP memristors are calculated to be 197 and 2150, respectively. These ON/OFF ratios were relatively greater than the value obtained from devices without any passivation (Figure S4). The operational stability of the memristors was further considered by testing the devices through endurance and retention testing. The endurance cycling test for 1000 cycles measured by voltage list sweep function (1 cycle = −5 V, +0.1 V, +4 V, and +0.1 V) is shown in Figure 2c. The resistance values at LRS for both PhNa-1T and PhNa-1T/OABr devices remained below 10^2^ Ω over 1000 cycles. However, the resistance value of the PhNa-1T device showed 10^3^ Ω, whereas PhNa-1T/OABr preserved a resistance level of close to 10^5^ Ω after 1000 cycles. For the retention testing of the OIHP memristors, as shown in Figure 2d, a continuous bias of 0.1 V was applied for 10^5^ s for both LRS and HRS. Both OIHP memristors with PhNa-1T and PhNa-1T/OABr showed consistency in the resistance values (below 10^2^ Ω) after 10^5^ s at LRS. The PhNa-1T device showed a resistance value of around 10^4^ Ω after 10^5^ s, whereas the PhNa-1T/OABr device resulted resistance value of close to 10^5^ Ω. Lastly, resistance values of 40 individual devices were recorded to draw cumulative probability distribution as shown in Figure S5. PhNa-1T interlayer-based OIHP memristors demonstrated σ/μ% of 32.1% while PhNa-1T/OABr devices showed 27.6%.

**Figure 3:**
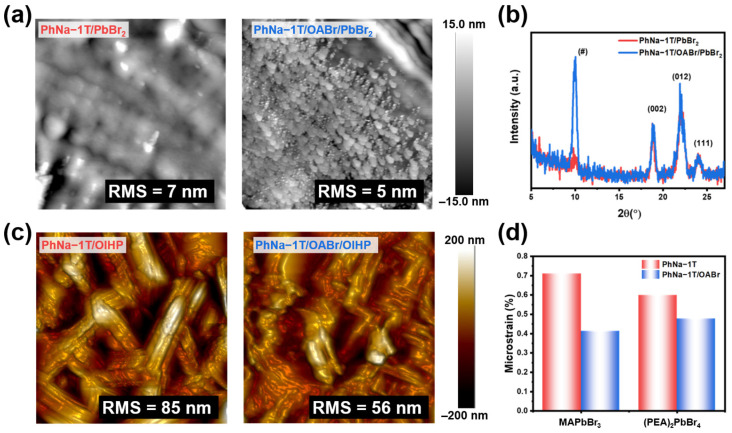
(**a**) AFM images and (**b**) XRD patterns for the PbBr_2_ films on PhNa−1T and PhNa−1T/OABr. (**c**) AFM images and (**d**) microstrain analysis for the OIHP films on PhNa−1T and PhNa−1T/OABr (AFM images were taken with dimensions of 4 μm × 4 μm).

To elucidate the reason for the improvement of meristic performance with PhNa-1T/OABr, the film investigation for the OIHP was examined using AFM and XRD measurements. Firstly, the topology of PbBr_2_ films on the interlayers was examined as shown in Figure 3a. The PbBr_2_ layer on the PhNa-1T film showed a smooth topology, with a root-mean-square (RMS) roughness value of 7 nm. Interestingly, distinct grains with an average size of 150 nm for PbBr_2_ films on PhNa-1T/OABr were observed with a slightly reduced RMS roughness value of 5 nm. The XRD patterns of the PbBr_2_ films were considered as shown in Figure 3b. A new strong peak at 2θ of 10° was observed for PbBr_2_ films on PhNa-1T/OABr without any further overall change in XRD peaks corresponding to PbBr_2_ films (Figure S6). The grains obtained from the PbBr_2_ AFM images would be OABr/PbBr_2_ adducts as shown by XRD data. Indifferent UV-vis absorption spectra for the PbBr_2_ films, as shown in Figure S7, imply that no OIHP is formed.

Then, the topology of the OIHP films was measured with concurrent measurement methodology after spin-coating ammonium salts on the PbBr_2_ layers. As shown in Figure 3c, the OIHP film on the PhNa-1T layer exhibited distinct rod-like grain structures with an RMS roughness value of 85 nm. The rod-like morphology of the OIHP films is concurrent with the reported 2D OIHPs [52,53]. In contrast, the OIHP film on the PhNa-1T/OABr layer displayed a more interconnected grain network with reduced rod-like features and a significantly lower RMS roughness value of 56 nm. This transformation indicates an improved grain connectivity and a smoother surface. Grain boundaries of OIHPs were reported to have relatively inferior memristic behavior than the intra-grain counterparts [54]. Moreover, a smoother surface can alleviate the formation of excessive CF during the operation, which is a source of the irreversible failure state of the OIHP memristors [55]. Furthermore, the presence of ammonium salt in CPE was known to assist in both interfacial passivation and OIHP morphology [56]. These morphological improvements would be associated with the OABr treatment on the CPE.

UV-vis absorption measurement and XRD, as shown in Figures S8 and S9, revealed that the OIHP layer is composed of mixed MAPbBr_3_ and (PEA)_2_PbBr_4_. Then, the peaks from the XRD measurement for OIHP films were analyzed by drawing Williamson–Hall plots for the derivation of microstrain (ε) of the crystallites, and the results are shown in Figure 3d [57]. The peaks for MAPbBr_3_ and (PEA)_2_PbBr_4_ were separately plotted. Crystallite sizes for MAPbBr_3_ with PhNa-1T were determined to be 11.0 nm, whereas PhNa-1T/OABr showed 13.8 nm. The absolute ε value for the OIHP layer on PhNa-1T was 7.10, and the PhNa-1T/OABr sample demonstrated a reduced value of 4.13. Similarly, (PEA)_2_PbBr_4_ crystallites showed a similar drop of ε value on PhNa-1T/OABr (4.77) than that on PhNa-1T (5.99) with similar crystallite sizes (15.2 and 14.7 nm for PhNa-1T and PhNa-1T/OABr, respectively). These results indicate that the growth of MAPbBr_3_ and (PEA)_2_PbBr_4_ crystallites are more favorable on PhNa-1T/OABr than on pristine PhNa-1T. Less strain of the crystallites would provide lower levels of defect [58]. The XPS data for Pb are in accordance with ε analysis, as shown in Figure S10. A slight reduction in metallic lead (Pb^0^) was observed for the OIHP films on PhNa-1T/OABr compared to films on PhNa-1T, as shown in Figure S11. Therefore, the reduced strains of the OIHP crystallites on PhNa-1T/OABr contribute to the higher resistance value at the HRS of corresponding OIHP-based memristors. In addition, the characteristics of OIHPs after the surface passivation were also investigated through UV-vis and XRD analysis, as shown in Figure S12.

**Figure 4:**
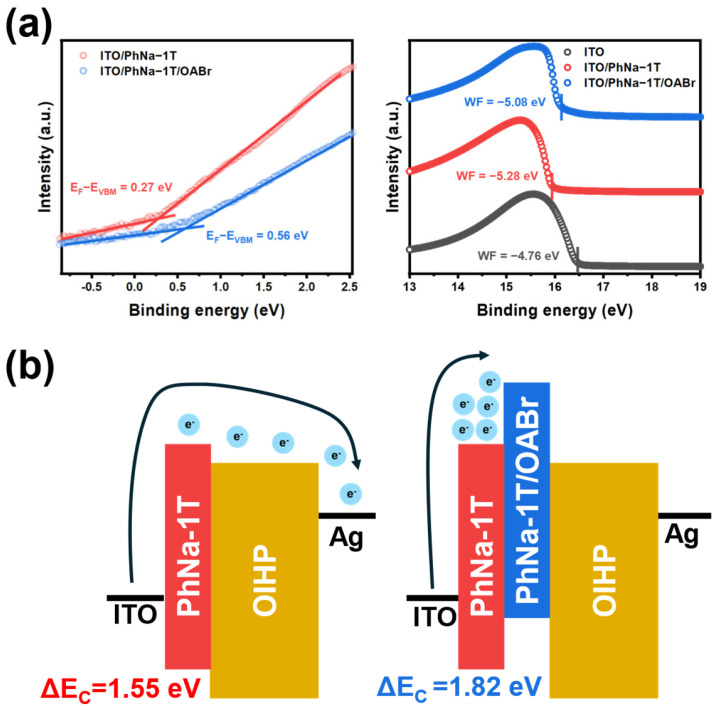
(**a**) UPS spectra for PhNa−1T and PhNa−1T/OABr films. (**b**) Schematic diagram for energy level alignments occurred for PhNa−1T and PhNa−1T/OABr.

Lastly, the energy level alignments of interlayers were investigated. Energy level alignments of different layers in a multi-stacked device are an important factor to be considered to modulate charge transport [59,60,61]. UPS measurements were conducted for PhNa-1T and PhNa-1T/OABr on ITO, as shown in Figure 4a. The work function calculated from bare ITO was 4.76 eV, which is within the range of the reported value of ITO [62]. The binding energy offset change when PhNa-1T and PhNa-1T/OABr were deposited on ITO was −0.52 and −0.32, respectively. This result indicates that the estimated Fermi energy (*E*_F_) of PhNa-1T is −5.28 eV and −5.08 eV for PhNa-1T/OABr. This upshift in the *E*_F_ would be due to the existence of ammonium ions on the interlayer surface [63,64]. The onset of the binding energy from UPS measurements for PhNa-1T was 0.27 eV, whereas 0.56 eV was found for PhNa-1T/OABr. This result is also consistent with the drop of polaron states in CPE upon the OABr treatment. Therefore, electrons moving from the ITO side to the counter electrode would experience a large energetic barrier to overcome in the device with PhNa-1T/OABr. We attribute such an enlarged energetic barrier as one of the main driving causes for the enhanced resistance value at the HRS. In addition, thermal activation energy (*E*_a_) was acquired from temperature-dependent current measurement according to the Arrhenius equation (ln (*I*) ~ exp(−*E*_a_/kT)). *I* refers to the current at −0.1 V, k is the Boltzmann constant, and T is the absolute temperature. The thermal *E*_a_ of 0.177 eV for PhNa-1T OIHP memristor was obtained while a higher *E*_a_ of 0.278 eV for PhNa-1T/OABr was observed, which is consistent with the enlarged energetic barrier at the interface between PhNa-1T/OABr and the OIHP layer, as shown in Figure S15.

Lastly, an additional energetic barrier induced from the surface passivation of OIHP would synergistically hamper band-to-band electron transport across the memristors. The surface energy level change upon surface treatment of OIHP was monitored through KPFM, as shown in Figure S13. A slight upshift (~150 mV) in the contact potential difference after the surface treatment was observed for both films on PhNa-1T and PhNa-1T/OABr. A detailed energy level alignment diagram for the layers adopted in this study is shown in Figure S14.

## 4. Conclusions

In summary, CPE was adopted as an interlayer between the BE and OIHP layers. The CPE layer was treated with OABr, which allowed the exchange of counter ions from Na to *n*-octyl ammonium. The self-doping characteristic of CPE was mitigated through the ion exchange, which resulted in the drop in conductance between BE and TE. The resulting OIHP memristors, along with surface passivation strategy with communal OABr treatment on OIHP, showed an improved ON/OFF ratio from 197 to 2150. The improvement was mainly derived from a drop in the resistance level at the HRS for the PhNa-1T interlayer after the OABr treatment. Further investigation of the OIHP layer formation on the PhNa-1T and PhNa-1T/OABr film demonstrated that the strain of perovskite crystallites is reduced when OABr is treated on CPE. Moreover, the OABr treatment of PhNa-1T produced an upshift in the energy level, providing the amplified energetical barrier for electron transport from BE to TE, which is consistent with the reduced current level at the HRS in the device with the PhNa-1T/OABr interlayer. We believe our findings provided by this study for ammonium salt treatment on CPE interlayer will contribute to the development of advanced optimization strategies for OIHP-based memristors.

## Data Availability

The data that support the findings of this study are available from the corresponding author upon reasonable request.

## Supplementary Materials

The following supporting information can be downloaded at: https://www.mdpi.com/article/10.3390/nano15030227/s1, Figure S1: *I–V* data; Figure S2: Electro-forming curves of OIHP; Figure S3: Linear fitting of current-voltage; Figure S4: Relative ON/OFF ratio with or without the surface passivation; Figure S5: Cumulative probability distribution data; Figure S6: XRD measurements; Figures S7 and S8: UV-vis absorption spectra; Figure S9: XRD plots on different interlayers; Figure S10: Williamson-Hall plots data; Figure S11: Pb XPS plots; Figure S12: UV-Vis absorption spectra and XRD patters after the surface passivation; Figure S13: KPFM measurements; Figure S14: UPS measurements and energy level diagram; Figure S15: Arrhenius plot data.

## References

[1] Jeong D.S., Thomas R., Katiyar R.S., Scott J.F., Kohlstedt H., Petraru A., Hwang C.S. (2012). Emerging memories: Resistive switching mechanisms and current status. Rep. Prog. Phys..

[2] Sun K., Chen J., Yan X. (2020). The future of memristors: Materials engineering and neural networks. Adv. Funct. Mater..

[3] Li X.-D., Chen N.-K., Wang B.-Q., Niu M., Xu M., Miao X., Li X.-B. (2024). Resistive Memory Devices at the Thinnest Limit: Progress and Challenges. Adv. Mater..

[4] Yang M., Zhao X., Tang Q., Cui N., Wang Z., Tong Y., Liu Y. (2018). Stretchable and conformable synapse memristors for wearable and implantable electronics. Nanoscale.

[5] Kim M.H., Park H.-L., Kim M.-H., Jang J., Bae J.-H., Kang I.M., Lee S.-H. (2021). Fluoropolymer-based organic memristor with multifunctionality for flexible neural network system. npj Flex. Electron..

[6] Lv Z., Zhu S., Wang Y., Ren Y., Luo M., Wang H., Zhang G., Zhai Y., Zhao S., Zhou Y. (2024). Development of bio-voltage operated humidity-sensory neurons comprising self-assembled peptide memristors. Adv. Mater..

[7] Leng Y.-B., Lv Z., Huang S., Xie P., Li H.-X., Zhu S., Sun T., Zhou Y., Zhai Y., Li Q. (2024). A near-infrared retinomorphic device with high dimensionality reservoir expression. Adv. Mater..

[8] Choi J., Park S., Lee J., Hong K., Kim D.-H., Moon C.W., Park G.D., Suh J., Hwang J., Kim S.Y. (2016). Organolead halide perovskites for low operating voltage multilevel resistive switching. Adv. Mater..

[9] John R.A., Shah N., Vishwanath S.K., Ng S.E., Febriansyah B., Jagadeeswararao M., Chang C.-H., Basu A., Mathews N. (2021). Halide perovskite memristors as flexible and reconfigurable physical unclonable functions. Nat. Commun..

[10] Kim S.J., Im I.H., Baek J.H., Choi S., Park S.H., Lee D.E., Kim J.Y., Kim S.Y., Park N.-G., Lee D. (2025). Linearly programmable two-dimensional halide perovskite memristor arrays for neuromorphic computing. Nat. Nanotechnol..

[11] Liu S., Li J., Xiao W., Chen R., Sun Z., Zhang Y., Lei X., Hu S., Kober-Czerny M., Wang J. (2024). Buried interface molecular hybrid for inverted perovskite solar cells. Nature.

[12] Fan P., Peng H.-X., Zheng Z.-H., Chen Z.-H., Tan S.-J., Chen X.-Y., Luo Y.-D., Su Z.-H., Luo J.-T., Liang G.-X. (2019). Single-source vapor-deposited Cs_2_AgBiBr_6_ thin films for lead-free perovskite solar cells. Nanomaterials.

[13] Tang Y., Jin P., Wang Y., Li D., Chen Y., Ran P., Fan W., Liang K., Ren H., Xu X. (2023). Enabling low-drift flexible perovskite photodetectors by electrical modulation for wearable health monitoring and weak light imaging. Nat. Commun..

[14] Wang H., Sun Y., Chen J., Wang F., Han R., Zhang C., Kong J., Li L., Yang J. (2022). A review of perovskite-based photodetectors and their applications. Nanomaterials.

[15] Abiram G., Thanihaichelvan M., Ravirajan P., Velauthapillai D. (2022). Review on perovskite semiconductor field–effect transistors and their applications. Nanomaterials.

[16] Sakhatskyi K., John R.A., Guerrero A., Tsarev S., Sabisch S., Das T., Matt G.J., Yakunin S., Cherniukh I., Kotyrba M. (2022). Assessing the drawbacks and benefits of ion migration in lead halide perovskites. ACS Energy Lett..

[17] Lee J.-Y., Lee S., Ryu J., Kang D.-W. (2024). Bandgap engineering via doping strategies for narrowing the bandgap below 1.2 eV in Sn/Pb binary perovskites: Unveiling the role of Bi^3+^ incorporation on different A-site compositions. Nanomaterials.

[18] Kim G.Y., Senocrate A., Wang Y.-R., Moia D., Maier J. (2021). Photo-effect on ion transport in mixed cation and halide perovskites and implications for photo-demixing. Angew. Chem..

[19] Zai H., Ma Y., Chen Q., Zhou H. (2021). Ion migration in halide perovskite solar cells: Mechanism, characterization, impact and suppression. J. Energy Chem..

[20] Tao S., Schmidt I., Brocks G., Jiang J., Tranca I., Meerholz K., Olthof S. (2019). Absolute energy level positions in tin- and lead-based halide perovskites. Nat. Commun..

[21] Shooshtari M., Traves M.J., Pahlavan S., Serrano-Gotarredona T., Linares-Barranco B. Applying Hodgkin-huxley neuron model for perovskite memristor in circuit simulation. Proceedings of the 2024 IEEE International Conference on Metrology for eXtended Reality, Artificial Intelligence and Neural Engineering (MetroXRAINE).

[22] Jiang B., Chen X., Pan X., Tao L., Huang Y., Tang J., Li X., Wang P., Ma G., Zhang J. (2024). Advances in metal halide perovskite memristors: A review from a co-design perspective. Adv. Sci..

[23] Li C., Hu M., Li Y., Jiang H., Ge N., Montgomery E., Zhang J., Song W., Davila N., Graves C.E. (2018). Analogue signal and image processing with large memristor crossbars. Nat. Electron..

[24] Perez-Martinez J.C., Berruet M., Gonzales C., Salehpour S., Bahari A., Arredondo B., Guerrero A. (2023). Role of metal contacts on halide perovskite memristors. Adv. Funct. Mater..

[25] Xu Z., Liu Z., Huang Y., Zheng G., Chen Q., Zhou H. (2017). To probe the performance of perovskite memory devices: Defects property and hysteresis. J. Mater. Chem. C.

[26] Lee J.H., Shim E.S., Kim Y.E., Jo J.W. (2024). Surface engineering of FAPbI3 based organic–inorganic hybrid perovskite for memristors. Appl. Phys. Lett..

[27] Gonzales C., Guerrero A., Bisquert J. (2021). Spectral properties of the dynamic state transition in metal halide perovskite-based memristor exhibiting negative capacitance. Appl. Phys. Lett..

[28] Opoku H., Kim Y.H., Lee J.H., Ahn H., Lee J.-J., Baek S.-W., Jo J.W. (2021). A tailored graft-type polymer as a dopant-free hole transport material in indoor perovskite photovoltaics. J. Mater. Chem. A.

[29] Yang X., Luo M., Zhang Q., Huang H., Yao Y., Yang Y., Li Y., Cheng W., Li P. (2024). Simultaneously enhancing the efficiency and stability of perovskite solar cells by using P3HT/PEDOT:PSS as a double hole transport layer. Nanomaterials.

[30] Lee J.H., Kim D., Opoku H., Ahn H., Lee J.-J., Baek S.W., Jo J.W. (2023). Ethylene glycol-containing ammonium salt for developing highly compatible interfaces in perovskite solar cells. Chem. Eng. J..

[31] Lee J.H., Kim T.H., Shim J.W., Jo J.W. (2024). A simply prepared amine oxide molecule as a low work function cathode modifier in organic photovoltaics for harvesting ambient light. J. Power Sources.

[32] Rhee R., Im S., Lee H., Lee J.H., Kim Y., Chun D.H., Park C., Lee S., Kim J.H., Park J.H. (2020). Stretchable hole extraction layer for improved stability in perovskite solar cells. ACS Sustain. Chem. Eng..

[33] Choi H., Mai C.-K., Kim H.-B., Jeon J., Song S., Bazan G.C., Kim J.Y., Heeger A.J. (2015). Conjugated polyelectrolyte hole transport layer for inverted-type perovskite solar cells. Nat. Commun..

[34] Jo J.W., Seo M.-S., Park M., Kim J.-Y., Park J.S., Han I.K., Ahn H., Jung J.W., Sohn B.H., Ko M.J. (2016). Improving performance and stability of flexible planar-heterojunction perovskite solar cells using polymeric hole-transport material. Adv. Funct. Mater..

[35] Lee B.H., Lee J.-H., Jeon S.Y., Park S.B., Lee S.H., Lee K. (2014). Broad work-function tunability of p-type conjugated polyelectrolytes for efficient organic solar cells. Adv. Energy Mater..

[36] Lee B.H., Jung I.H., Woo H.Y., Shim H.-K., Kim G., Lee K. (2013). Multi-charged conjugated polyelectrolytes as a versatile work function modifier for organic electronic devices. Adv. Funct. Mater..

[37] Chen Z., Hu Z., Wu Z., Liu X., Jin Y., Xiao M., Huang F., Cao Y. (2017). Counterion-tunable n-type conjugated polyelectrolytes for the interface engineering of efficient polymer solar cells. J. Mater. Chem. A.

[38] Jung E.D., Harit A.K., Kim D.H., Jang C.H., Park J.H., Cho S., Song M.H., Woo H.Y. (2020). Multiply charged conjugated polyelectrolytes as a multifunctional interlayer for efficient and scalable perovskite solar cells. Adv. Mater..

[39] Kim S., Jeong I., Park C., Kang G., Han I.K., Kim W., Park M. (2019). Morphology control of perovskite in green antisolvent system for MAPbI_3_-based solar cells with over 20% efficiency. Sol. Energy Mat. Sol. C..

[40] Kheirabadi H., Abdizadeh H., Golobostanfard M.R. (2021). Boosting the graded structure of 2D perovskite solar cell based on BA_2_MA*_n_*_–1_Pb*_n_*I_3*n*+1_ by noninteger *n* values. ACS Appl. Energy Mater..

[41] Wan Y., Zhang X., Bazan G.C., Nguyen T.-Q., Lu G. (2022). Polarons in conjugated polyelectrolytes: A first-principles perspective. Adv. Funct. Mater..

[42] Yoo J.J., Wieghold S., Spnseller M.C., Chua M.R., Bertram S.N., Hartono N.T.P., Tresback J.S., Hansen E.C., Correa-Baena J.-P., Bulovic V. (2019). An interface stabilized perovskite solar cell with high stabilized efficiency and low voltage loss. Energy Environ. Sci..

[43] Loizos M., Tountas M., Mangelis P., Rogdakis K., Kymakis E. (2023). Surface passivation of sequentially deposited perovskite solar cells by octylammonium spacer cations. APL Energy.

[44] Danielsen S.P.O., Thompson B.J., Fredrickson G.H., Nguyen T.-Q., Bazan G.C., Segalman R.A. (2022). Ionic Tunability of Conjugated Polyelectrolyte Solutions. Macromolecules.

[45] Panzer F., Bassler H., Kohler A. (2017). Temperature induced order–disorder transition in solutions of conjugated polymers probed by optical spectroscopy. J. Phys. Chem. Lett..

[46] Mai C.-K., Schlitz R.A., Su G.M., Spitzer D., Wang X., Fronk S.L., Cahill D.G., Chabinyc M.L., Bazan G.C. (2014). Side-chain effects on the conductivity, morphology, and thermoelectric properties of self-doped narrow-band-gap conjugated polyelectrolytes. J. Am. Chem. Soc..

[47] Lee J.H., Shim E.S., Nketia-Yawson B., Opoku H., Ahn H., Bae S., Jo J.W. (2024). Suppressed surface aggregation and homogeneous integration of π-Bridged polyelectrolyte for boosting charge transport in conjugated polymer semiconductors. Appl. Surf. Sci..

[48] Burschka J., Pellet N., Moon S.-J., Humphry-Bakr R., Gao P., Nazeeruddin M.K., Gratzel M. (2013). Sequential deposition as a route to high-performance perovskite-sensitized solar cells. Nature.

[49] Liu Y., Li F., Chen Z., Guo T., Wu C., Kim T.W. (2016). Resistive switching memory based on organic/inorganic hybrid perovskite materials. Vacuum.

[50] Lee S., Wolfe S., Torres J., Yun M., Lee J.-K. (2021). Asymmetric bipolar resistive switching of halide perovskite film in contact with TiO_2_ layer. ACS Appl. Mater. Interfaces.

[51] Xia F., Xu Y., Li B., Hui W., Zhang S., Zhu L., Xia Y., Chen Y., Huang W. (2020). Improved performance of CH_3_NH_3_PbI_3−x_Cl_x_ resistive switching memory by assembling 2D/3D perovskite heterostructures. ACS Appl. Mater. Interfaces.

[52] Lai H., Kan B., Liu T., Zheng N., Xie Z., Zhou T., Wan X., Zhang X., Liu Y., Chen Y. (2018). Two-dimensional ruddlesden–popper perovskite with nanorod-like morphology for solar cells with efficiency exceeding 15%. J. Am. Chem. Soc..

[53] Huang F., Siffalovic P., Li B., Yang S., Zhang L., Nadazdy P., Cao G., Tian J. (2020). Controlled crystallinity and morphologies of 2D Ruddlesden-Popper perovskite films grown without anti-solvent for solar cells. Chem. Eng. J..

[54] Heo J.H., Shin D.H., Moon S.H., Lee M.H., Kim D.H., Oh S.H., Jo W., Im S.H. (2017). Memory effect behavior with respect to the crystal grain size in the organic-inorganic hybrid perovskite nonvolatile resistive random access memory. Sci. Rep..

[55] Ren Y., Ma H., Wang W., Wang Z., Xu H., Zhao X., Liu W., Ma J., Liu Y. (2018). Cycling-induced degradation of organic–inorganic perovskite-based resistive switching memory. Adv. Mater. Technol..

[56] Li X., Wang Y.-C., Zhu L., Zhang W., Wang H.-Q., Fang J. (2017). Improving efficiency and reproducibility of perovskite solar cells through aggregation control in polyelectrolytes hole transport layer. ACS Appl. Mater. Interfaces.

[57] Canchanya-Huaman Y., Mayta-Armas A.F., Pomalaya-Velasco J., Bendezu-Roca Y., Guerra J.A., Ramos-Guivar J.A. (2021). Strain and grain size determination of CeO_2_ and TiO_2_ nanoparticles: Comparing integral breadth methods versus rietveld, μ-raman, and TEM. Nanomaterials.

[58] Cheng H., Liu C., Zhuang J., Cao J., Wang T., Wong W.-Y., Yan F. (2022). KBF_4_ Additive for alleviating microstrain, improving crystallinity, and passivating defects in inverted perovskite solar cells. Adv. Funct. Mater..

[59] Lee J.H., Nketia-Yawson B., Lee J.-J., Jo J.W. (2022). Ionic liquid-mediated reconstruction of perovskite surface for highly efficient photovoltaics. Chem. Eng. J..

[60] Sabbah H., Baki Z.A. (2023). Device simulation of highly stable and 29% efficient FA_0.75_MA_0.25_Sn_0.95_Ge_0.05_I_3_ -based perovskite solar cell. Nanomaterials.

[61] Kim T.H., Lee J.H., Jang M.H., Lee G.M., Shim E.S., Oh S., Saeed M.A., Lee M.J., Yu B.-S., Hwang D.K. (2024). Atto-scale noise near-infrared organic photodetectors enabled by controlling interfacial energetic offset through enhanced anchoring ability. Adv. Mater..

[62] Fattahi A., Koohsari P., Lakmehsari M.S., Ghandi K. (2022). The impact of the surface modification on tin-doped indium oxide nanocomposite properties. Nanomaterials.

[63] Xia R., Leem D.-S., Kirchartz T., Spencer S., Murphy C., He Z., Wu H., Su S., Cao Y., Kim J.S. (2013). Investigation of a conjugated polyelectrolyte interlayer for inverted polymer:fullerene solar cells. Adv. Energy Mater..

[64] Yao J., Qiu B., Zhang Z.-G., Xue L., Wang R., Zhang C., Chen S., Zhou Q., Sun C., Yang C. (2020). Cathode engineering with perylene-diimide interlayer enabling over 17% efficiency single-junction organic solar cells. Nat. Commun..

