# Supplementary material for: Effect of Ammonium Salt on Conjugated Polyelectrolyte as an Interlayer for Organic–Inorganic Hybrid Perovskite Memristors
Source: Nanomaterials (Basel). 2025 Jan 30;15(3):227. doi: 10.3390/nano15030227 (PMC11819950; doi:10.3390/nano15030227)
Supplement: Supplementary file 1 [file nanomaterials-15-00227-s001.zip › nanomaterials-3429769-supplementary.pdf]

Effect of Ammonium Salt on Conjugated Polyelectrolyte as an Interlayer for Organic-inorganic Hybrid Perovskite Memristors

Eun Soo Shim†, Ji Hyeon Lee†, Ju Wan Park, Sun Woo Kim, Su Bin Park and Jea Woong Jo*

Department of Energy and Materials Engineering, Dongguk University, 30 Pildong-ro, 1-gil, Jung-gu, Seoul 04620, South Korea

* Correspondence: whwp78@dongguk.edu

† These authors contributed equally to this work

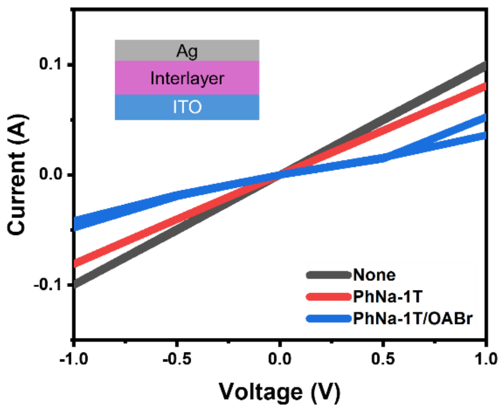

Figure S1. *I–V* data of ITO / Interlayer / Ag.

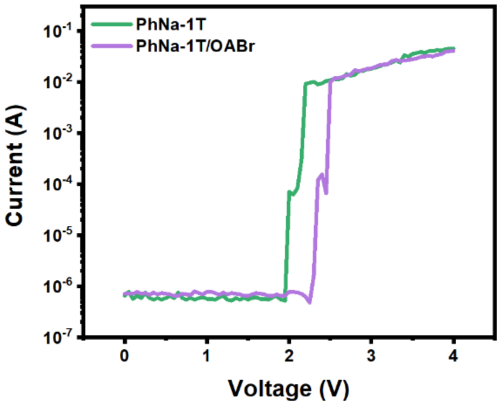

Figure S2. Electro-forming curves of the OIHP memristors.

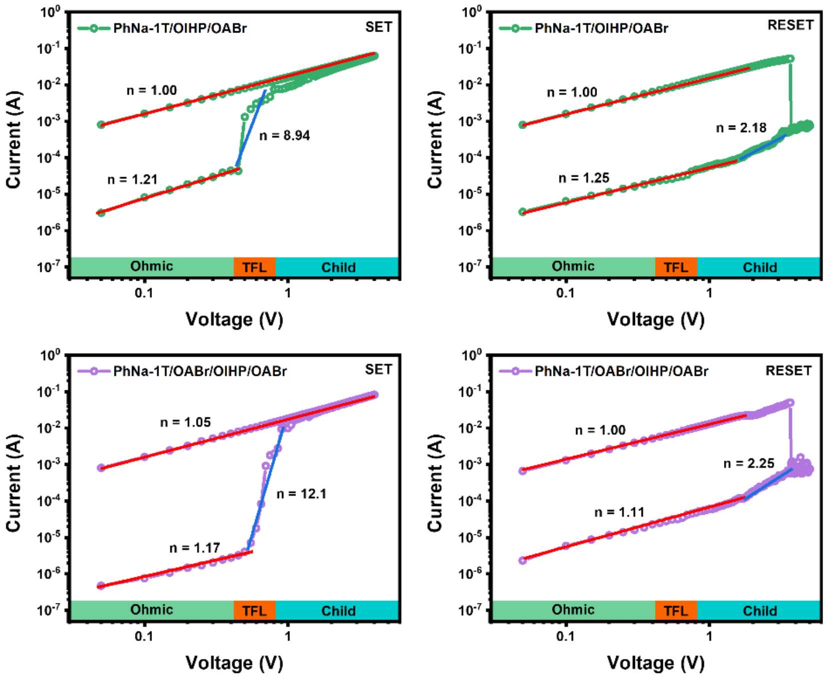

Figure S3. Linear fitting of current-voltage in log-log scale for SET, and RESET processes from OIHP memristors.

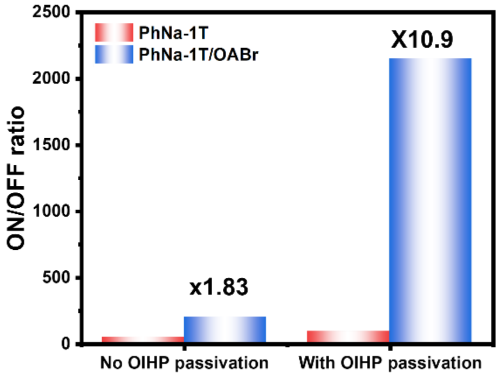

Figure S4. Relative ON/OFF ratio for the interlayer-based memristors with or without the surface passivation of OIHP

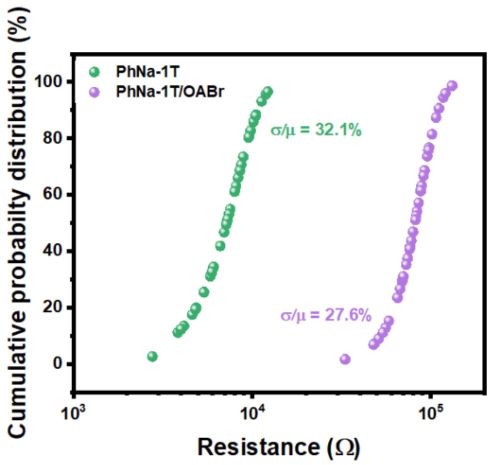

Figure S5. Cumulative probability distribution data for the PhNa-1T and PhNa-1T/OABr based OIHP memristors.

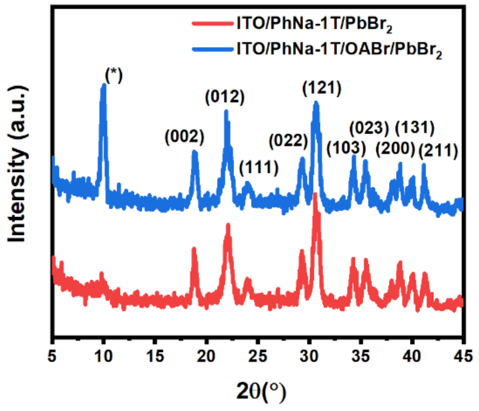

Figure S6. XRD patterns of the $PbBr_2$ films on different interlayers

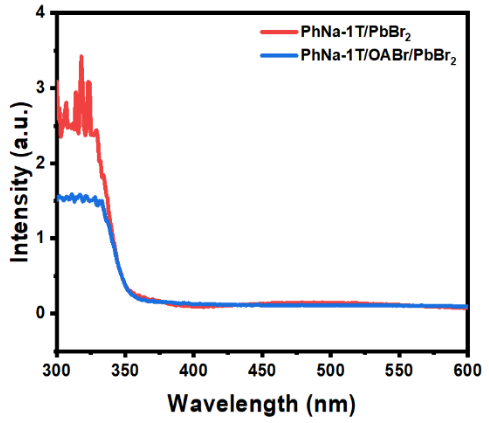

Figure S7. UV-vis absorption spectra of the $PbBr_2$ films on different interlayers

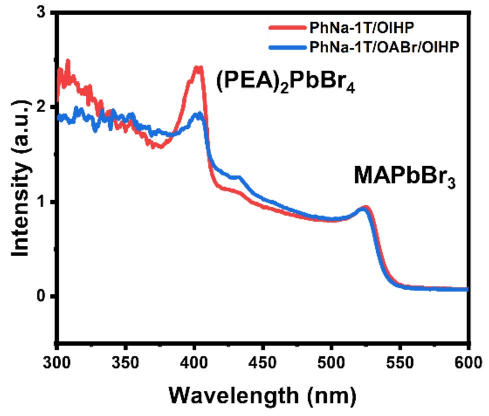

Figure S8. UV-vis absorption spectra of the OIHP films on different interlayers

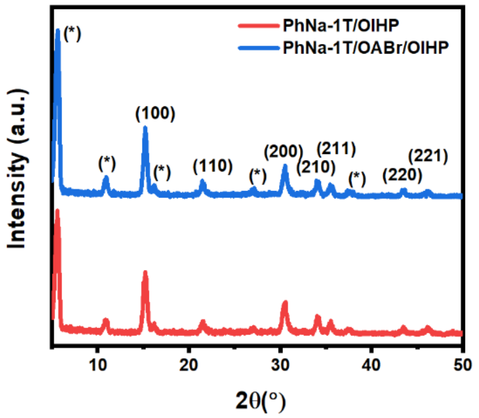

Figure S9. XRD plots of the OIHP films on different interlayers (* indicate peaks corresponding to ($(PEA)_2PbBr_4$)

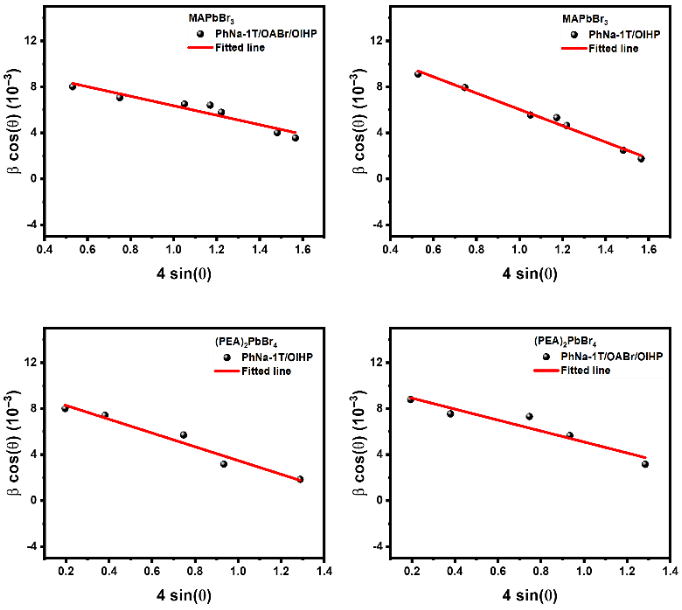

Figure S10. Williamson-Hall plots for the $MAPbBr_3$, and $(PEA)_2PbBr_4$ films on different interlayers

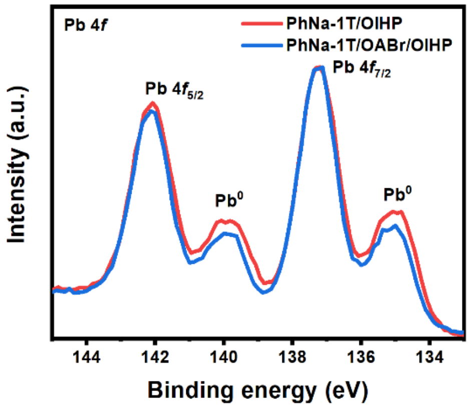

Figure S11. Pb XPS plots for the OIHP films on different interlayers

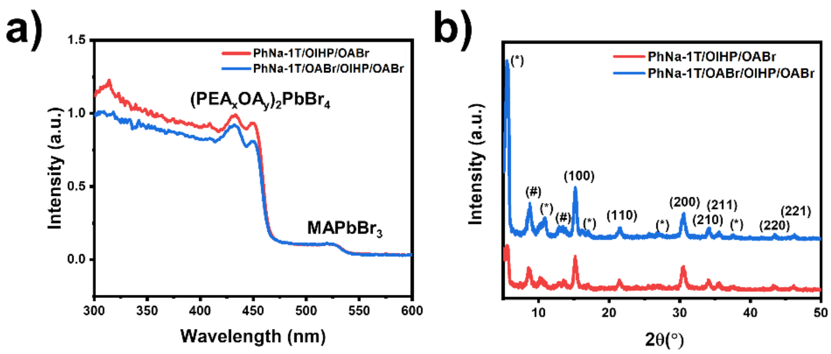

Figure S12. (a) UV-Vis absorption spectra, and (b) XRD patterns for the OIHP films after the surface passivation (* indicates peaks for $(PEA)_2PbBr_4$, and # for $(OA_xPEA_y)_2PbBr_4$).

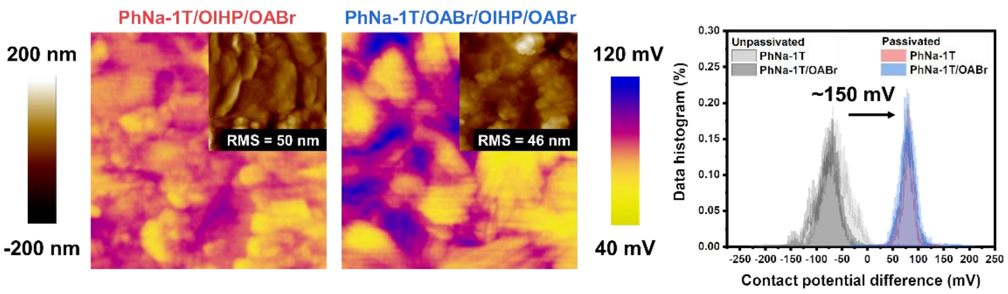

Figure S13. KPFM measurements for the OIHP layers after the surface passivation.

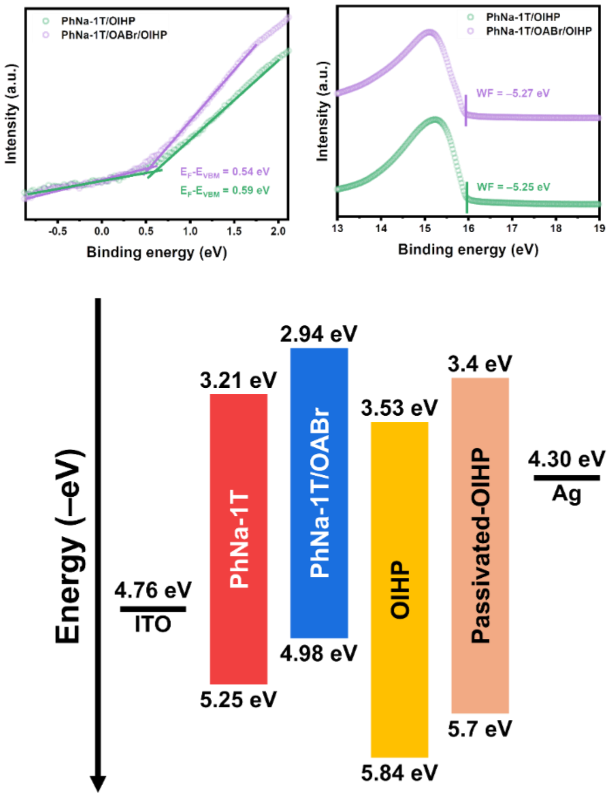

Figure S14. UPS measurement for the OIHP layers on different interlayers. Energy level diagram of layers used in this study.

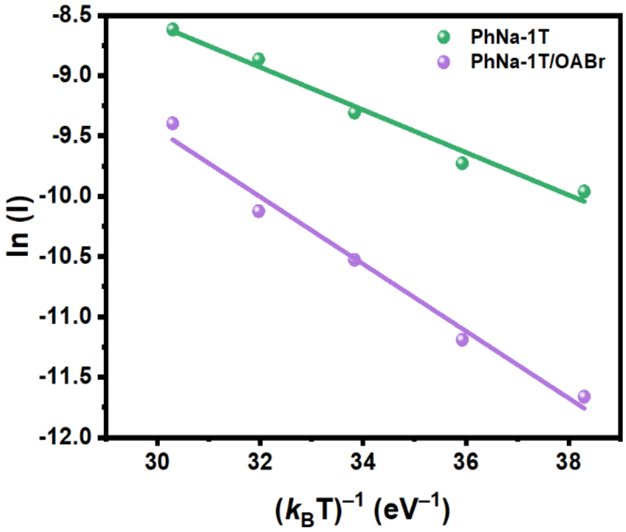

Figure S15. Arrhenius plot of OIHP memristors depending on different interlayer.
